# A Synthetic Lethal Screen Identifies a Role for *Lin-44*/Wnt in *C*. *elegans* Embryogenesis

**DOI:** 10.1371/journal.pone.0121397

**Published:** 2015-05-04

**Authors:** Samantha N. Hartin, Martin L. Hudson, Curtis Yingling, Brian D. Ackley

**Affiliations:** 1 Department of Molecular Biosciences, University of Kansas, Lawrence, KS, United States of America; 2 Department of Biology and Physics, Kennesaw State University, Kennesaw, GA, United States of America; Brown University/Harvard, UNITED STATES

## Abstract

**Background:**

The *C*. *elegans* proteins PTP-3/LAR-RPTP and SDN-1/Syndecan are conserved cell adhesion molecules. Loss-of-function (LOF) mutations in either *ptp-3* or *sdn-1* result in low penetrance embryonic developmental defects. Work from other systems has shown that syndecans can function as ligands for LAR receptors *in vivo*. We used double mutant analysis to test whether *ptp-3* and *sdn-1* function in a linear genetic pathway during *C*. *elegans* embryogenesis.

**Results:**

We found animals with LOF in both *sdn-1* and *ptp-3* exhibited a highly penetrant synthetic lethality (SynLet), with only a small percentage of animals surviving to adulthood. Analysis of the survivors demonstrated that these animals had a synergistic increase in the penetrance of embryonic developmental defects. Together, these data strongly suggested PTP-3 and SDN-1 function in parallel during embryogenesis. We subsequently used RNAi to knockdown ~3,600 genes predicted to encode secreted and/or transmembrane molecules to identify genes that interacted with *ptp-3* or *sdn-1*. We found that the Wnt ligand, *lin-44*, was SynLet with *sdn-1*, but not *ptp-3*. We used 4-dimensional time-lapse analysis to characterize the interaction between *lin-44* and *sdn-1*. We found evidence that loss of *lin-44* caused defects in the polarization and migration of endodermal precursors during gastrulation, a previously undescribed role for *lin-44* that is strongly enhanced by the loss of *sdn-1*.

**Conclusions:**

PTP-3 and SDN-1 function in compensatory pathways during *C*. *elegans* embryonic and larval development, as simultaneous loss of both genes has dire consequences for organismal survival. The Wnt ligand *lin-44* contributes to the early stages of gastrulation in parallel to *sdn-1*, but in a genetic pathway with *ptp-3*. Overall, the SynLet phenotype provides a robust platform to identify *ptp-3* and *sdn-1* interacting genes, as well as other genes that function in development, yet might be missed in traditional forward genetic screens.

## Introduction

Cell adhesion molecules (CAMs) provide multiple functions during the development and homeostasis of an organism. In *C*. *elegans*, multiple CAMs contribute to early embryonic development and loss-of-function (LOF) mutations in these can result in cellular, tissue and/or organismal abnormalities [1–4]. Interestingly, these molecules often appear to act in semi-redundant ways, where input from multiple CAMs are required for the fidelity of a specific developmental event [3, 5–7]. This can be best observed when LOF in a single gene has a modest effect on viability, but LOF in two genes in combination can have severe effects leading to highly penetrant lethality or arrest. This synergistic effect, known as Synthetic Lethality (SynLet), can be harnessed to uncover genetic interactions between functional pathways [8–14].

We and others have previously described developmental defects associated with *ptp-3*, the *C*. *elegans* Leukocyte-common antigen related (LAR)-like receptor protein tyrosine phosphatase (RPTP) [5, 7, 15–17]. LAR is a member of the type IIa family of tyrosine phosphatases [18]. Vertebrates have three type IIa family members; LAR (PTPRF), RPTPσ (PTPRD) and RPTPδ (PTPRS) [19]. LAR-like RPTPs have been implicated in multiple aspects of nervous system development [20–27]. LAR is also required for proper mammary gland development in mice [28] and the LAR genomic locus is frequently deleted in breast, colon, and other cancers of epithelial origin [29]. Together, the pleiotropic nature of these observations highlights the importance of LAR-like receptor tyrosine phosphatases in organismal development and homeostasis.

LAR-like RPTPs are receptors for extracellular matrix molecules, including laminin, chondroitin sulfate proteoglycans and heparan sulfate proteoglycans (CSPGs and HSPGs, respectively) [24, 30–32]. In *Drosophila* DLAR has been shown to bind syndecan and glypican, two cell-surface associated HSPGs [33, 34], consistent with reports from vertebrates demonstrating that LAR binds HSPG molecules [30, 32]. LAR family members have also been shown to physically and genetically interact with HSPGs in Zebrafish trigeminal and Rohon-Beard neuron development [35]. Importantly, *Drosophila* genetic studies, in addition to mammalian sensory neuron explant assays demonstrate that competition between distinct HSPG/CSPG ligands for LAR-like RPTPs can exert opposing effects on neural development [32, 34].

Syndecans are cell-surface associated HSPGs that have been implicated in a broad range of developmental events, and have been linked to the modulation of several secreted morphogens including Wnts and Fibroblast Growth Factors (FGFs) [36–43]. The extracellular domain of syndecans can be post-translationally modified with HS- or CS-side chains and the intracellular domain can interact with cytoplasmic signaling effectors via a PDZ binding motif. The *C*. *elegans* genome encodes a single syndecan, SDN-1, which, by sequence, is most similar to human syndecan-2 (SDC2) [44–46].

Here we provide genetic evidence that *ptp-3B* and *sdn-1* function in parallel signaling pathways during *C*. *elegans* embryonic development. *ptp-3; sdn-1* double mutants exhibit a highly penetrant SynLet phenotype, with development arresting during embryogenesis or in the first larval stage (L1). A small percentage of animals do progress to adulthood, but exhibit sterility or low fecundity with all offspring arresting during development.

Using an RNAi library comprised primarily of predicted secreted proteins, we screened for genes that exhibited a SynLet phenotype with worms homozygous for either *ptp-3* or *sdn-1* LOF mutations. From a screen of 3,652 clones, we isolated 25 candidate SynLet genes, and several additional candidate genes displayed an increase in lethality or slow growth phenotype in either the *ptp-3* or *sdn-1* background, but did not meet our threshold criteria for synthetic lethality. Among these, we found that the Wnt ligand, *lin-44*, was strongly SynLet with *sdn-1*, but not *ptp-3*. Using a time-lapse microscopy approach we found defects in the ingression of the endodermal precursor cells Ea and Ep in *lin-44* mutants. This phenotype was significantly enhanced when *sdn-1* was also removed. This is the first data indicating that LIN-44 contributes to gastrulation events in *C*. *elegans* and demonstrates the power of using double mutant analyses to uncover novel roles for well-characterized genes.

## Materials and Methods

### Genetics

The following alleles were used in this report: N2 (var. Bristol), *sdn-1(zh20)*, *sdn-1(ok449)*, *ptp-3(mu254)*, *ptp-3(mu256)*, *ptp-3A(ok244)*, *ptp-3(op147)*, *lin-17(n671)*, *lin-44(n1792)*, *eri-1(mg366)*, *juIs76* [*Punc-25*::*gfp*], *jcIs1* [P*ajm-1*::AJM-1::GFP] and *mIn1mIs14*. Strains were maintained at 18–22°C, using standard maintenance techniques as described [47]. All lethality counts were conducted with animals maintained at 20°C.

To score the synthetic lethality of the *ptp-3; sdn-1* double mutant animals we generated *sdn-1(zh20)* homozygotes where *ptp-3*(*mu245)*, was balanced with a chromosomal inversion, *mIn1*, which is marked with a recessive mutation (*dpy-10)*, and a dominant pharyngeal GFP insertion, *mIs14* (phGfp). We further marked the *mu245* lesion by linking it to a GABAergic neuronal marker, *juIs76* [*Punc-25*::*gfp*] (nGfp). Progeny from the *sdn-1(zh20); ptp-3(mu245)juIs76/mIn1mIs14* mothers were expected to define the following three phenotypic classes and their corresponding genotypes:

- Dpy+phGfp—(*sdn-1(zh20); mIn1mIs14/mIn1mIs14*)
- phGfp+nGfp+NonDpy—*(sdn-1(zh20); ptp-3(mu245)juIs76/mIn1mIs14)*
- NonDpy+NonphGfp+nGfp—*sdn-1(zh20); ptp-3(mu245)juIs76*.

### RNAi feeding control strains used

RNAi clones were compiled from the Ahringer library [48]. Three RNAi controls were used during each round of screening, an empty vector (L4440), and clones targeting *ptp-3* (II-7J03; Overlapping CDS: C09D8.1) or *sdn-1* (pEVL202). The *sdn-1* fragment designated for RNAi was obtained by polymerase chain reaction (PCR) from N2 genomic DNA. The fragment was then cloned into the pCR8 TOPO cloning vector (Life Technologies) and recombined via an LR reaction into a dual T7-feeding vector derived from the L4440 vector (a kind gift of A. Fire). The resulting plasmid was transformed into the HT115(DE3) RNase III-deficient *E*. *coli* strain [49]. The following primer pairs were used for PCR amplification of *sdn-1*: forward 5’-TTTTCTTTTAGAACCCTTTTGC-3’ reverse: 3’-CATCAATTTATCATCTCGCAAC-5’.

### RNAi feeding assay (6-well format)

HT115 bacterial strains, containing the RNAi clones of interest, were grown overnight at 37°C in 1.5 mL LB plus ampicillin, tetracycline and nystatin. 0.1 ml of the overnight cultures were aliquoted on single 6-well plates of NGM containing carbenicillin, tetracycline, and 1mM IPTG and grown overnight at 37°C. Approximately 3–4 L4 worms were dispensed in M9 into the top wells (1, 2 and 3) on the 6-well plates. The plates were left at approximately 20°C for five days before the worms were scored for phenotypes and 3–4 L4 worms were transferred to the bottom wells (4, 5 and 6). The bottom wells were then scored after six more days. The experimenter was blind to the RNAi clone being tested in all assays. All clones that exhibited any level of SynLet were rescreened as described above to verify the interaction. Clones were then rescreened if they displayed SynLet or slow growth with any of the query strains. We categorized SynLet as embryonic lethal (Emb), larval lethal (Lvl) or adult lethal (Adl). The following additional phenotypes were also scored: Lethal (Let), body morphology defects (Bmd), uncoordinated (Unc), sickness (Sck), sterility (Ste), slow growing (Gro), protruding vulva (Pvl), dumpy (Dpy) and/or egg laying defective (Egl). All phenotypes were compared between the strains on that 6-well plate alone.

### RNAi clone sequencing

Clones isolated from the screen were grown as single clones in liquid LB cultures containing ampicillin, tetracycline and nystatin. Cultures were purified using a Qiagen spin mini-prep kit then sequenced using a modified forward T7 primer (5’-ACTCACTATAGGGAGACCGG-3’).

### Time-lapse microscopy of embryonic development

4-dimensional video microscopy was carried out using an Olympus BX61 microscope equipped with a 63x magnification oil immersion objective and motorized z-axis stage. Z-stacks (27–35 slices) were collected every 2 minutes at 1 micron spacing using a Retiga camera (Q-Imaging). Data sets were analyzed via the Bioformats-Importer and 4D Browser plugins in ImageJ [50, 51]. The following time points were collected; Ea/Ep ingression, gastrulation cleft opening, cleft closure, and comma stage. Analysis of developmental time points was normalized relative to Ea/Ep ingression and comma stage. Embryonic lethal phenotypes were categorized according to [52]. Ea/Ep ingression behavior was scored as follows; I, normal (Ea/Ep ingress together); II, no ingression (Ea/Ep remain on the outer surface of the embryo); III, skewed (either Ea or Ep ingresses before its partner); IV, unclassified (Ea/Ep migration obscured or unclear).

### Lethality analysis

A single L4 hermaphrodite was placed on a single NGM plate to initiate the assay. Every 24 hours the hermaphrodite was transferred to a new plate. 24 hours after the removal of the hermaphrodite the plate was analyzed for embryos (Emb) or dead L1 larvae (Lva). The plate was rescreened 24 hours later for later larval lethality (Lva). Adults were transferred until they died, or until they stopped giving rise to offspring. All animals were maintained at 20°C, except during scoring. Experimenter was blind to the genotype during scoring.

### Epithelial morphology assays

Epithelial tight junctions were visualized with *jcIs1 [P-ajm-1-AJM-1*::*GFP]*, which localizes to epithelial junctions, essentially outlining all epithelial cells from about the lima bean stage onwards. Multiple embryos were harvested by bleaching and > 100 assayed for epithelial morphology. Embryos with cells that were obviously mispositioned, (*e*.*g*. dorsal cells on the ventral side), or misshapen in a way that was strikingly different from wild-type, (*e*.*g*. rounded cells along the lateral aspect where cuboidal cells are normally present), were scored as a mutant.

## Results and Discussion

### Dual loss of ptp-3 and sdn-1 results in synthetic lethality

**Fig 1:**
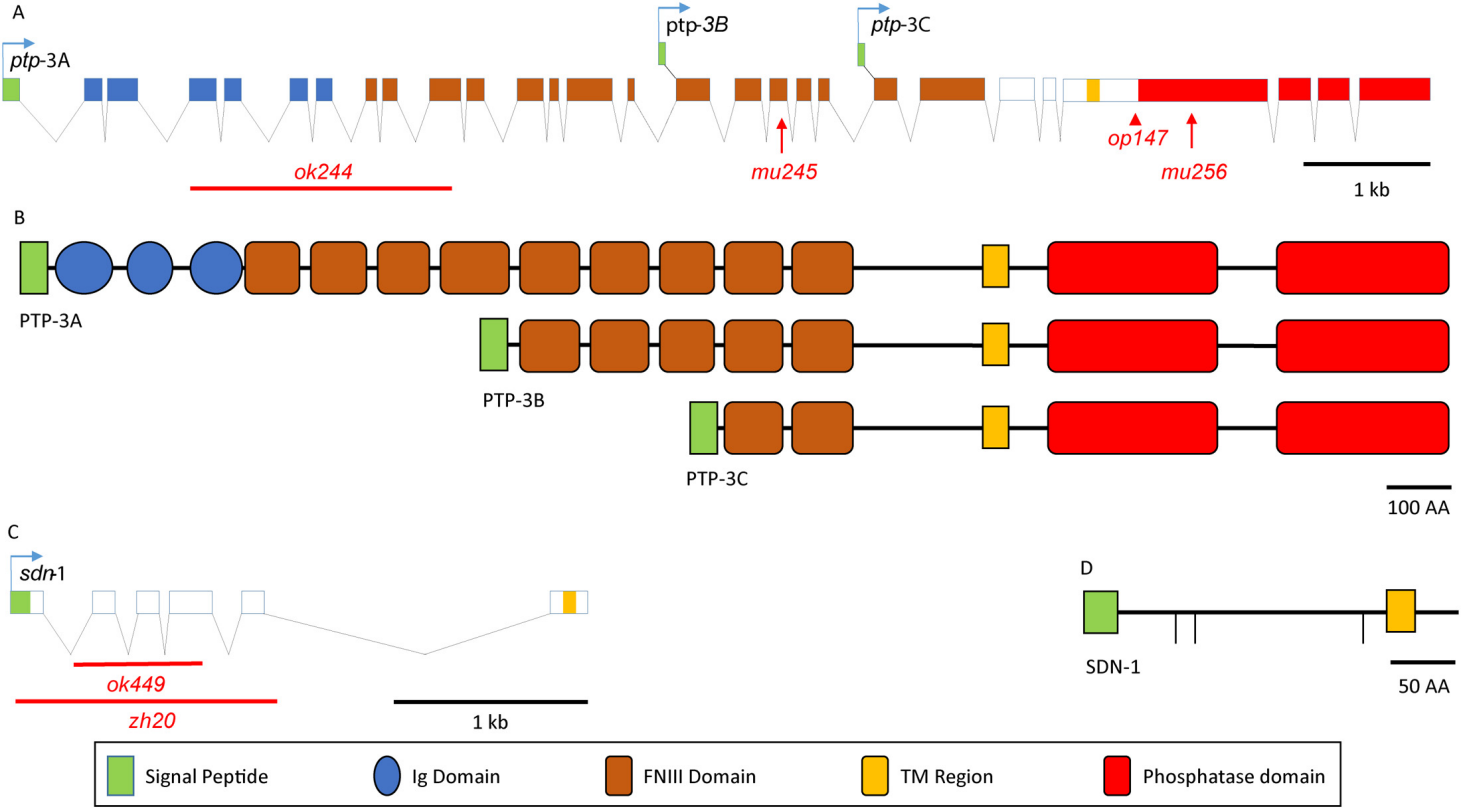
Genomic and protein structure of *ptp-3* and *sdn-1*. A schematic of the gene and protein structures of *ptp-3* and *sdn-1* are presented with lesions used in this report indicated. A) The *ptp-3* genomic locus gives rise to at least three independently generated transcripts, *ptp-3A*, *ptp-3B* and *ptp-3C*. The *ptp-3(ok244)* deletion specifically affects *ptp-3A*, *ptp-3(mu245)* is a premature stop that affects *ptp-3A* and *ptp-3B*, *ptp-3(op147)* is a Tc1 transposon insertion and *ptp-3(mu256)* is a frameshift and premature stop that affects all three transcripts. B) The three PTP-3 protein isoforms differ by the composition of the extracellular domains. See key at bottom of figure for domain architecture. C) The *sdn-1* genomic locus produces a single transcript. The two deletion alleles, *sdn-1(zh20)* and *sdn-1(ok449)*, both remove a large portion of the *sdn-1* coding segment and are strong loss of function alleles. D) The SDN-1 protein has three SG motifs (vertical lines) that are predicted to be targets for the addition of heparan sulfate side chains.

**Fig 2:**
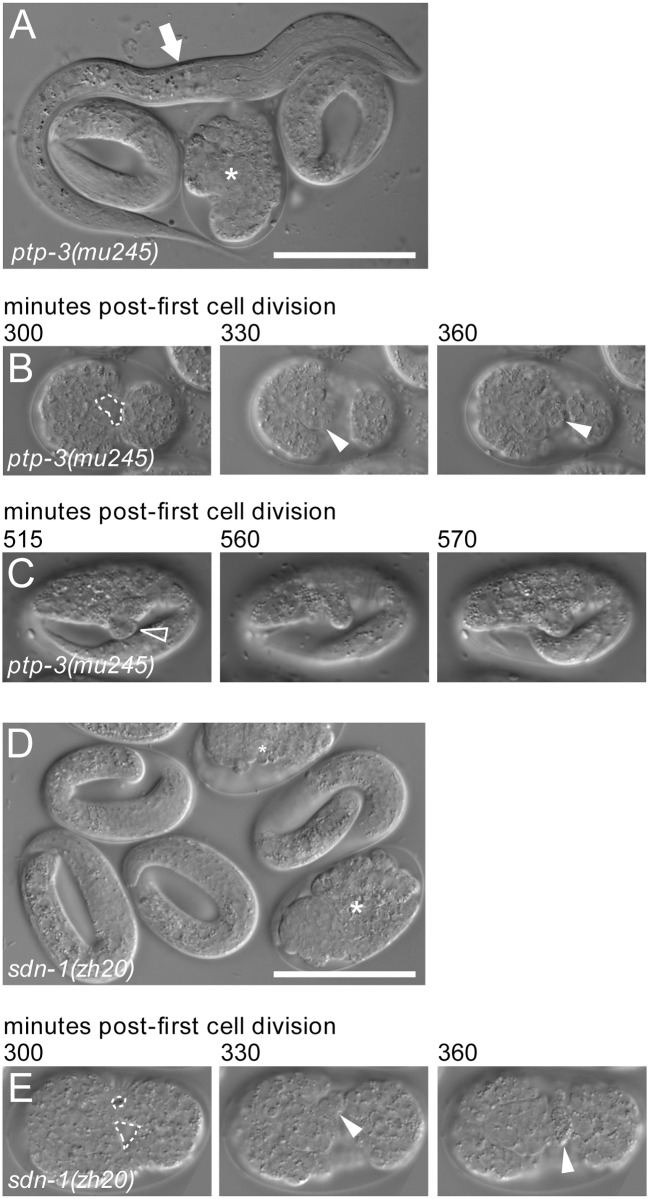
Loss of function in *ptp-3* and *sdn-1* results in low penetrance embryonic and larval defects. A: *ptp-3(mu245)* mutant animals can hatch as normal looking L1 larvae (arrow), but can also die during embryogenesis (asterisk shows an embryo that ruptured at elongation). B: *ptp-3* mutants can exhibit defects in ventral neuroblast migration (B1 outlined area), which result in a persistent gastrulation cleft on the ventral surface. When these fail to close, internal cells extrude through the opening (arrowheads) during elongation, leading to embryonic lethality. C: *ptp-3* mutants can also exhibit variably abnormal herniations in body morphology (empty arrowhead). D: *sdn-1(zh20)* mutants also exhibit low penetrance embryonic lethality (asterisks show ruptured embryos). E: Gastrulation cleft closure defects are observed in some *sdn-1(zh20)* mutant embryos (outlined area). Again, this can lead to embryonic rupture at ventral enclosure (arrowhead shows cells oozing from within).

Both *ptp-3* and *sdn-1* have previously described roles in *C*. *elegans* embryonic development [5, 7, 44, 53]. The *ptp-3* locus encodes three distinct transcripts, each with independent promoters [15] (Fig 1). Mutations in *ptp-3B* exhibit a low level of embryonic (Emb) and larval lethality (Lva) as well as Variable-abnormal body morphology defects (Vab) (Fig 2 and Table 1). However, most *ptp-3* LOF mutants are superficially normal in appearance. Similarly, *sdn-1* mutants exhibit low levels of Emb and Lva offspring (Table 1), but most grow to adulthood, where they exhibit uncoordinated movement (Unc) and egg-laying defects (Egl).

**Table 1:** Lethality by genotype analyses.

Genotype ^a^	Brood Size Avg (St Dev) ^b^	Phenotype ^c^ Avg (St Dev)				N ^d^
		Emb	Lva	Vab	WT	
wild type	247.6 (35.6)	0.5 (0.6)	1.0 (0.7)	0.0 (0.0)	98.5 (0.9)	1238
*ptp-3(mu245)*	82.0 (51.6)	2.1 (1.7)	8.4 (4.7)	2.2 (2.8)	87.3 (7.2)	984
*sdn-1(zh20)*	173.6 (101.0)	7.7 (5.3)	5.5 (3.7)	0.6 (0.7)	86.6 (6.1)	868
*ptp-3(mu245)/mIn1mIs14; sdn-1(zh20)*	114.2 (35.7)	8.2 (2.6)	9.6 (1.8)	1.8 (2.4)	80.4 (4.2)	1142
*ptp-3(mu245); sdn-1(zh20)*	10.2 (6.7)	84.3 (8.4) †	15.7 (4.1) †	0.0 (0.0)	0.0 (0.0) †	51
*lin-44(n1792)*	137.4 (33.9)	12.2 (2.9)	10.6 (2.1)	0.4 (0.6)	76.8 (3.4)	687
*sdn-1(zh20); lin-44(n1792)*	94.8 (38.4)	71.6 (11.0) † ‡	4.6 (1.9)	0.4 (0.9)	23.5 (12.0) † ‡	474
*sdn-1(zh20); lin-17(n671)*	76.6 (12.6)	4.4 (8.0)	1.3 (1.1)	0.0 (0.0)	93.6 (7.8)	383
*ptp-3(mu245); lin-44(n1792)*	101.2 (49.0)	4.6 (3.2) ‡	21.5 (5.7) ‡	1.3 (1.3)	68.7 (8.7) ‡	506

^*a*^ Listed genotype is the maternal genotype.

^*b*^ average total brood size of five mothers.

^*c*^ percent of animals displaying phenotype from mothers of indicated genotype.

^*d*^ total number of offspring analyzed.

^*†*^ Significantly different from *sdn-1(zh20)* p<0.01 (Students t-test).

^‡^ Significantly different from *lin-44(n1792)* p<0.01 (Students t-test).

Based on the low penetrance viability defects in *ptp-3* mutants, and previous observations that the Drosophila LAR receptor, DLAR, can bind syndecan [33, 34], we asked whether *ptp-3* and *sdn-1* could be interacting genetically during *C*. *elegans* development. If PTP-3 and SDN-1 were acting as a ligand-receptor pair, we would have expected that *ptp-3; sdn-1* double mutants would exhibit embryonic lethality at a rate similar to the single mutant genetic backgrounds. In contrast we found that animals lacking both *sdn-1* and *ptp-3* were essentially inviable (Table 1). To score the development of these animals we generated *sdn-1(zh20)* homozygotes where a strong *ptp-3* LOF mutation, *mu245*, was balanced with a chromosomal inversion, *mIn1*, which is marked with a recessive mutation (*dpy-10)*, and a dominant pharyngeal GFP insertion, *mIs14* (phGfp) (see Materials and methods).

We found an increase in the embryonic and larval lethality in offspring from *sdn-1(zh20); ptp-3(mu245)juIs76/mIn1mIs14* mothers compared to *sdn-1(zh20); juIs76* or *ptp-3(mu245)juIs76* alone. Compared to the expected 25% of the brood, only 12.6% of live hatchlings were *ptp-3; sdn-1* (126/1000 offspring from a total of 5 mothers). Further, when these broods were analyzed on the first day of adulthood, only 2% (2/114 offspring) of the adults were *sdn-1(zh20); ptp-3(mu245)juIs76*). Surviving *sdn-1(zh20); ptp-3(mu245)juIs76* adults appeared sickly, were largely paralyzed and had no viable offspring (78 Emb and 5 Lva L1s from 4 mothers). SynLet phenotypes were also observed when we tested other strong *ptp-3* LOF mutations including *ptp-3(mu256)* and *ptp-3(op147)*, and a second deletion allele in *sdn-1*, *ok449*. None of the *ptp-3; sdn-1* double mutant combinations were viable as homozygous strains, and each had to be maintained as balanced *ptp-3*/*mIn1mIs14* heterozygotes for propagation. In addition, RNAi targeting *sdn-1* was lethal in *ptp-3(mu245)* animals, and RNAi targeting *ptp-3* was lethal in *sdn-1(zh20)* animals. These data confirm that the SynLet phenotypes observed are due to the alleles in question and not caused by closely linked background mutations.

We more closely analyzed the *sdn-1(zh20); ptp-3(mu245)* homozygous animals to determine the point at which they were arresting. We found instances of early embryonic arrest (pre-morphogenesis), embryonic rupture during epidermal enclosure, and arrest at the L1 stage as misshapen larvae. These are similar arrest points to those observed in embryos from *sdn-1(zh20); ptp-3(mu245)/mIn1mIs14* mothers. However, the higher rate of embryonic arrest in the offspring of *sdn-1(zh20); ptp-3(mu245)* animals than from *sdn-1(zh20); ptp-3(mu245)/mIn1mIs14* mothers strongly suggests a maternal contribution of *ptp-3* to embryonic development. Since the offspring of *sdn-1*(*zh20); ptp-3(mu245)* animals, which should lack any maternal contribution, demonstrated a variable arrest point, we concluded there are likely several stages of development to which both SDN-1 and PTP-3 contribute, in partially compensatory ways. However, we cannot completely discount that defects that occur early in development may present as variable arrest phenotypes. Overall these results indicate that *ptp-3* and *sdn-1* have overlapping function during embryogenesis and that loss of both genes results in dire consequences for organismal survival.

We previously demonstrated that two of the isoforms produced by the *ptp-3* locus have differential localization [5, 15]. PTP-3A localization is restricted to synapses, while PTP-3B is associated with cell-cell junctions during embryogenesis but also localizes to axons during axon outgrowth. Consistent with their different localizations, PTP-3A and PTP-3B appear to function as distinct genetic units as loss of *ptp-3A* has no embryonic patterning or axon guidance defects, yet exhibits a fully penetrant synaptic morphology defect that is equivalent to a complete loss of function for the *ptp-3* locus [15]. Similarly, PTP-3B is capable of rescuing the embryonic development, cell migration, and axon outgrowth defects associated with *ptp-3* LOF mutations [1, 5, 17, 54]. The *ptp-3(mu245)* lesion specifically affects the PTP-3A and PTP-3B isoforms, and we did not observe a SynLet phenotype when we used a LOF mutation in *ptp-3* that specifically affects the PTP-3A isoform, *ptp-3A(ok244)* (Fig 1). This is consistent with our previous results suggesting that PTP-3A does not obviously contribute to epidermal development, while PTP-3B does.

**Fig 3:**
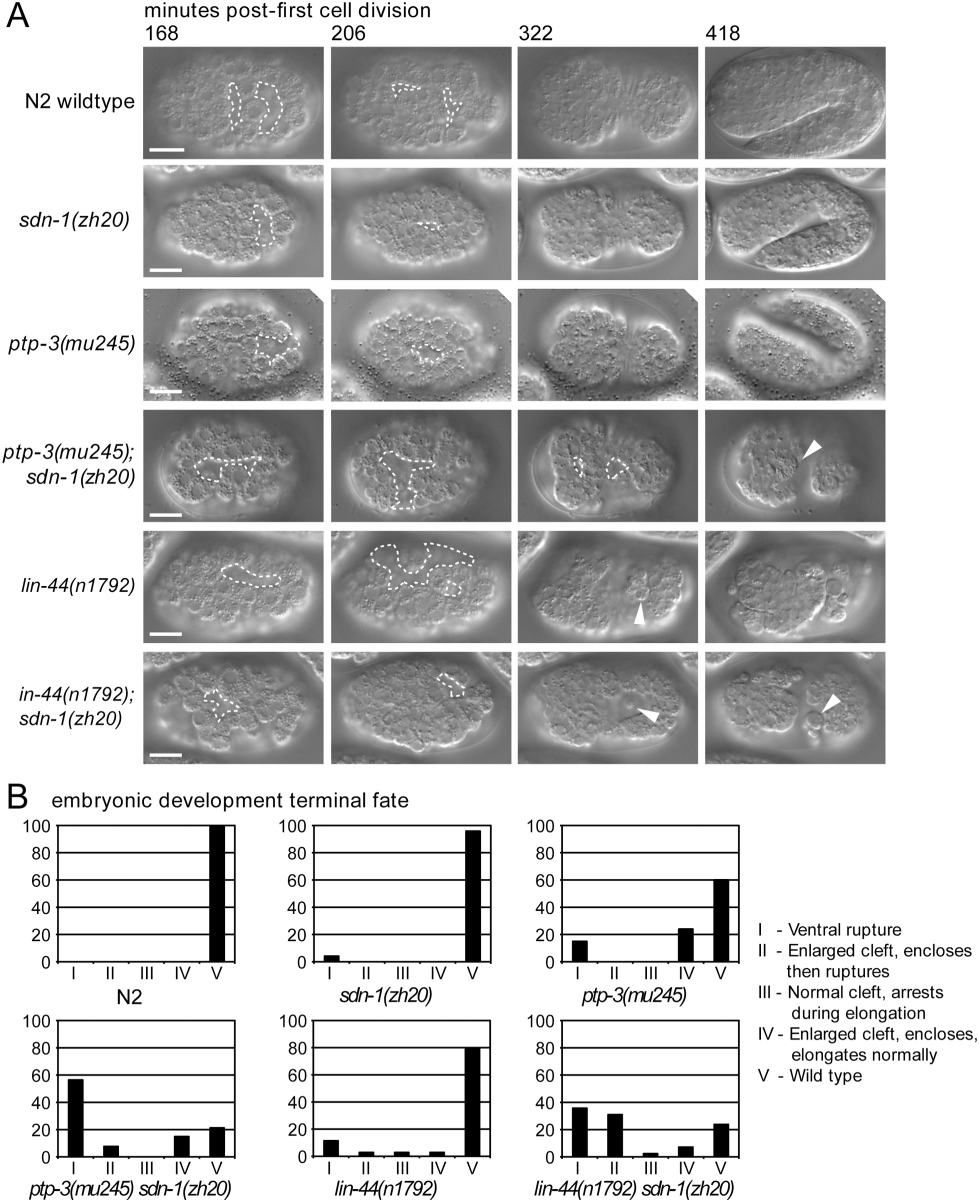
The dual loss of *ptp-3* and *sdn-1* results in synergistic defects during embryogenesis. A: Using 4D time-lapse microscopy we monitored embryogenesis in N2 wild-type, *ptp-3*, *sdn-1*, *lin-44* single mutants, and *lin-44; sdn-1* and *ptp-3; sdn-1* double mutants. The gastrulation clefts (outlined regions) present in the single mutants are more likely to close during development than the clefts in the double mutants. The arrowhead indicates cells that have extruded from the internal region of the embryo through the open gastrulation cleft (panel 418 minutes). B: Terminal fates of the embryos plotted by genotype.

To better understand what might be contributing to *ptp-3; sdn-1* lethality during embryogenesis, we used 4D time-lapse microscopy to observe cell division and migration during the first 10 hours of embryonic development. The first cell migration event that occurs during *C*. *elegans* development is the onset of gastrulation, where the gut precursor cells, Ea and Ep, ingress into the center of the embryo [55]. In wild-type embryos, Ea and Ep ingress in concert, side-by-side, with the space vacated by their ingression filled by movements from six surrounding cells [56]. The next major developmental event occurs when cells of the endodermal and mesodermal lineages begin to ingress at the posterior end, leaving a transient gastrulation cleft on the ventral surface (Fig 3) [57]. Onset of cleft opening was phenotypically normal in the embryos observed (n = 10) although one embryo showed gastrulation cleft opening at the anterior end. However, the relative timing of gastrulation cleft opening was significantly delayed in *ptp-3* embryos when compared to Ea/Ep ingression and comma stage. The gastrulation cleft is flanked by neuroblasts; in wild-type embryos, these normally migrate towards the midline of the ventral surface, closing the cleft in about 55 minutes [53]. These subsequently form a substrate for epithelial cell migration, which intercalate and extend from the dorsal and lateral surfaces to enclose the embryo during epiboly.

In *ptp-3; sdn-1* mutants, 8/14 embryos showed gastrulation clefts that persisted until epithelial extension and ventral enclosure (Fig 3). All of these embryos ruptured prior to comma stage, at the onset of embryonic elongation (class I phenotype, [52]). Two large cells were frequently seen in the center of these enlarged clefts that showed no adhesion to the cells surrounding them. Based on previous cell lineaging experiments, we tentatively identified these as the germ line precursor cells Z2 and Z3 (M. L. Hudson, unpublished observations). Additional embryos showed gross disorganization during development, with lateral loss of cell—cell contacts on the embryo surface prior to cleft opening. In the embryos that survived ventral enclosure, defects were also observed in tail morphology. While gastrulation cleft opening appears to be significantly delayed in *ptp-3; sdn-1* mutants compared to *ptp-3* alone, only 3/10 *ptp-3; sdn-1* mutants could be scored for this phenotype as most embryos rupture prior to reaching comma stage, and hence cannot be scored for the final developmental timeline marker. As such, this apparent suppression of *ptp-3* developmental timing defects may be misleading. Overall, the most common cause of embryonic lethality was failure to close the gastrulation cleft prior to ventral enclosure. These data confirm previously identified roles for SDN-1 and PTP-3 in embryonic morphogenesis [5, 53], and suggest that SDN-1 and PTP-3 function in parallel, part-redundant pathways to control either cell adhesion, neuroblast migration or both.

### An RNAi screen for SynLet interactions with *ptp-3* and *sdn-1*

The observation that simultaneous LOF in *ptp-3* and *sdn-1* resulted in a highly penetrant SynLet phenotype suggested that we could use these backgrounds to identify additional genes that contribute to embryonic development [8]. We employed RNAi knockdown to systematically screen for genes that lead to a synergistic SynLet phenotype in either *ptp-3(mu256)* and/or *sdn-1(zh20)* mutant backgrounds. RNAi clones that were identified from the first round of screening were retested at least four times to confirm the results. The threshold for declaring an interaction as synthetic lethal was >75% Emb. We also identified genes that were SynLet with both *ptp-3* and *sdn-1*, suggesting these may function in yet another independent parallel pathway, or may contribute in overlapping fashion to both the *ptp-3* and *sdn-1* pathways.

We found 11 genes that showed a SynLet phenotype in *ptp-3(mu256)* animals, but only limited or no lethality in a wild type background (Table 2, S1 Table). Two of these genes, *vab-1* and *unc-40*, have previously been found to be SynLet with *ptp-3*, indicating that our screen was capable of identifying relevant genetic interactions [5, 7, 17, 46]. We also found genes that are known to be individually lethal via complete loss-of-function mutations, including *bli-3* and *mek-2*. However, in our assays, RNAi knockdown in the wild type background was insufficient to cause highly penetrant lethality. We conclude that this approach enabled us to identify genetic interactions that might be missed using traditional loss-of-function alleles.

**Table 2:** SynLet Genes by Genotype Affected.

WormBase Identification	Overlapping CDS(s)	Gene Name	Description
SynLet with *ptp-3*			
WBGene00006776	T19B4.7	*unc-40*	Netrin receptor
WBGene00007301	C04F12.7		Multiple transmembrane domains
WBGene00007768	C27C7.5		Carbohydrate binding domain protein
WBGene00023237	C17C3.14		Psuedogene
WBGene00006868	M03A1.1	*vab-1*	Ephrin receptor tyrosine kinase
WBGene00016638 ^a^	C44B12.5	*perm-4*	Secreted protein
WBGene00022539 ^a^	ZC190.5		Multiple transmembrane domains
SynLet with *sdn-1*			
WBGene00007900	C33D9.6		Coiled coil domains
WBGene00015009	B0041.5		Solute carrier family 35-like
WBGene00016753	C48E7.8	*oac-9*	O-acyltransferase
WBGene00008411	D2030.1	*mans-1*	Mannosidase
WBGene00000254	K04F10.4	*bli-4*	KEX2/subtilisin-like serine endoprotease
WBGene00020397	T10B11.1	*pcbd-1*	pterin-4-α-carbinolamine dehydratase
WBGene00009666	F43G9.3		Secreted protein
WBGene00006061	F30A10.5	*stl-1*	Stomatin-like
WBGene00011783	T15D6.9		Secreted protein
WBGene00016253	C30E1.4		Secreted protein
WBGene00003029 ^b^	E01A2.3	*lin-44*	Wnt ligand
SynLet with *ptp-3* and *sdn-1*			
WBGene00000253	F56C11.1	*bli-3*	Dual oxidase
WBGene00003569	F35C12.2	*ncx-4*	Na^+^/Ca^2+^ exchanger
WBGene00008294	C54C8.7	*clec-11*	C-type lectin
WBGene00003186	Y54E10BL.6	*mek-2*	MAP kinase kinase
*wt* Let, but not in *ptp-3* or *sdn-1*			
WBGene00044058	F17B5.6		Carbohydrate transferase
WBGene00007139	B0285.7	*mnp-1*	Matrix non-peptidase
WBGene00007666	C18B12.4		Plasma membrane-associated ring-finger domain containing protein

^a^ Let phenotype observed in wild-type also, but not in *sdn-1*.

^b^ Let phenotype observed in wild-type also, but not in *ptp-3*.

Seven of the genes found to be SynLet in the *ptp-3* background had an attenuated effect when knocked down in the *sdn-1* background (Table 2). These genes are candidates to function in *sdn-1* mediated development. Of these several have been associated with the formation or function of the nervous system (including *unc-40*, *vab-1*, *mek-2* and C27C7.5). Because syndecans are associated with neural development it suggests either failures in neural development can interfere with normal embryogenesis, or that these molecules perform non-neural developmental functions [2, 58]. A second theme that emerged when analyzing the list is that several of the genes have an association with gametogenesis or the germline. For example, *perm-4* is expressed in oocytes, and regulates an interaction with sperm, while the C04F12.7 gene is co-expressed with several sperm-specific genes. VAB-1 has also been linked to the function of germline maintenance [59]. Finally, the ZC190.5 has been previously identified as a suppressor of the *egl-9* locus [60]. EGL-9 encodes a proline hydroxylase that negatively regulates HIF-1 signaling [61], but also participates in neural development [62]. Thus, it will be interesting to determine whether the identification of these genes implicates syndecan in oxygen sensing or germline development/function.

We isolated 15 clones that generated a SynLet phenotype in *sdn-1* mutants, but had no effect, or an attenuated one, when knocked down in wild type animals. Of these, 11 of these were also less penetrant in *ptp-3* animals, suggesting they are candidates to function in *ptp-3*-dependent development. One of the most interesting candidates to emerge was the Wnt ligand, *lin-44* (see below). Wnt ligands contribute to multiple facets of organismal development throughout the animal kingdom. Interestingly, a recent paper describes an interaction between *sdn-1* and another *C*. *elegans* Wnt ligand, *mom-2*, where SDN-1 concentrates the MIG-5/Dishevelled protein in early embryogenesis [62].

We also identified several genes that may contribute to post-translational modifications of proteins, possibly including Wnts, such as an O-acyltransferase (*oac-9)*, a mannosidase (*mans-1)*, a pterin-4-α-carbinolamine dehydratase (*pcbd-1*), a putative sugar transporter (B0041.5), a subtilisin-like endoproteases (*bli-4*) and a dual-oxidase (*bli-3*). It should be possible to use the *lin-44—sdn-1* interaction we describe below to tease out potential contributions of these molecules to Wnt-dependent functions. Two of the genes are likely involved in mitochondrial function (*stl-1*, and F43G9.3), although it is unclear whether this function is contributing to the embryonic lethality when knocked down in *sdn-1*.

An unanticipated outcome of the RNAi SynLet screen was the discovery of three genes where RNAi was lethal to our control strain yet showed incomplete penetrance in *ptp-3* or *sdn-1* mutant backgrounds. Two of those genes, C18B12.4 and *mnp-1* are reported to cause lethality when mutated, or when knocked down in wild-type animals via RNAi [63–66]. The third gene, F17B5.6, is predicted to code for a glycosyl transferase and has not previously been reported to have a role in embryonic development. *mnp-1*, which encodes a 781 amino acid protein related to the M1 family of metalloproteinases, is required during embryonic development to facilitate muscle cell migrations from lateral to dorsal and ventral positions [66]. Previous work has demonstrated that *mnp-1* genetically interacts with the Eph Receptor *vab-1*, which is SynLet with *ptp-3*, suggesting that the interactions between these genes maybe more complicated than previously suggested. Interestingly, *mnp-1* is predicted to be catalytically inactive due to the lack of three of four essential zinc-binding amino acids, and thus may function in more of a structural role, perhaps by occluding peptidase sites to promote structural integrity.

In the course of our screen we found other genes that interacted genetically with *ptp-3* and/or *sdn-1*, but these interactions were either too variable, or did not repeat in multiple assays, to formally conclude their role in embryonic and larval development (S1 Table). Some of these caused slowed growth (Gro) or an apparent sickness (Sck) that lead to decreased viability over the experimental window, while others may have caused increased changes in the body morphology defects (Bmd or Vab) (S1 Table). Although these were not included in the list of SynLet candidates, the genes may merit analysis in the future when attempting to further understand how PTP-3 or SDN-1 contribute to morphogenesis.

### Epidermal junction defects do not correlate with SynLet phenotypes

PTP-3B is associated with cell junctions during the cellular migrations and tissue rearrangements that occur during embryogenesis, and *ptp-3B* mutants exhibit low-penetrance epidermal morphology (Vab) defects [5]. SDN-1 also localizes to the plasma membrane, being concentrated at cell-cell junctions in early embryos [62]. We hypothesized that lethality might arise from disruption of epidermal junction formation or patterning. To assay this we examined a marker for epidermal junctions, AJM-1::GFP, in *sdn-1* or *ptp-3* mutants alone and when grown on enhancer gene RNAi expressing bacteria.

**Fig 4:**
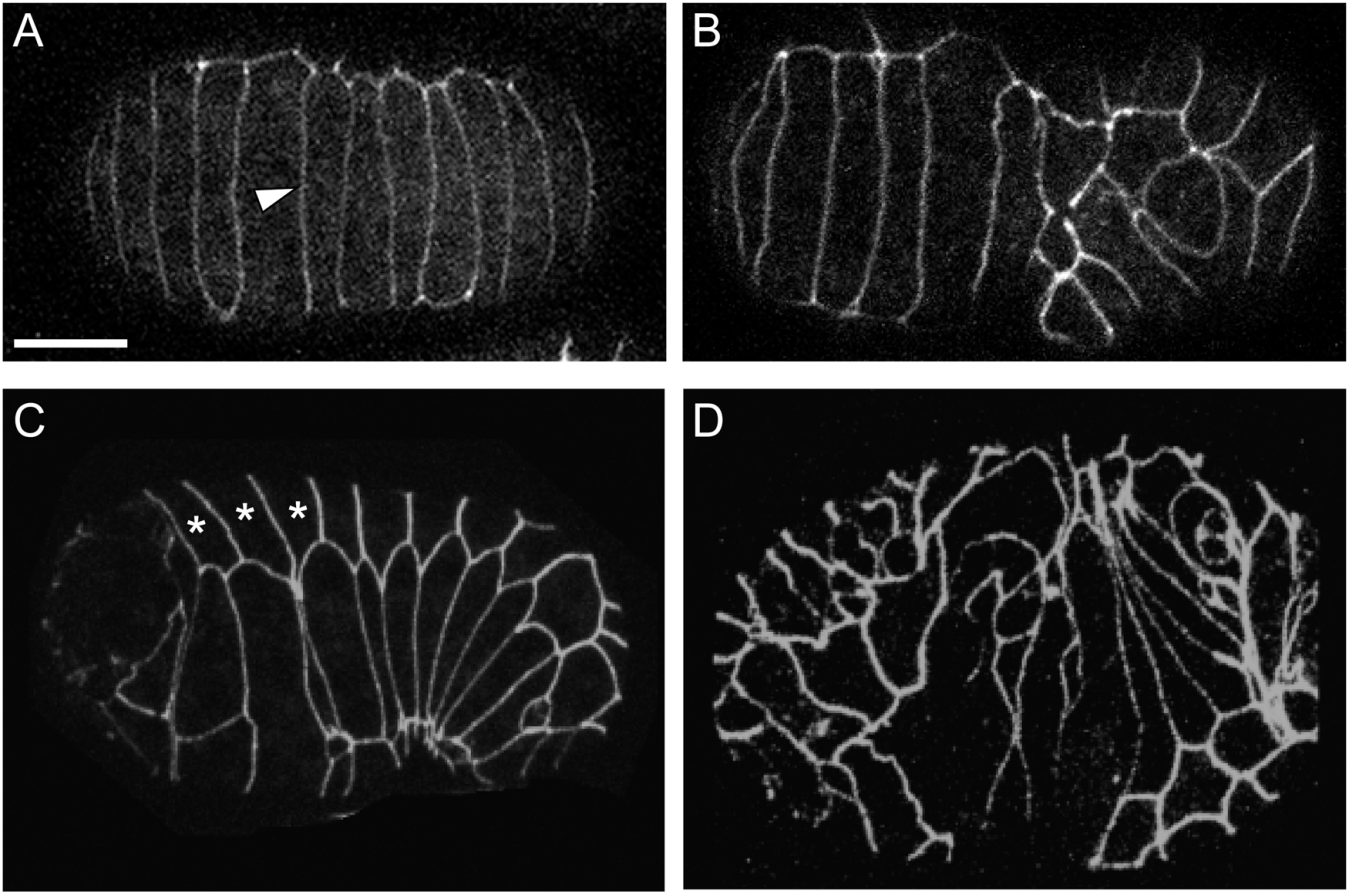
Epidermal junctions can be maintained in cell-adhesion mutants and RNAi treated animals. We used an AJM-1::GFP transgene (*jcIs1)* to examine epidermal morphology in animals being tested. A: In wild-type *jcIs1* animals, AJM-1::GFP is localized to cell junctions (arrow) and outlines epidermal cells. Here a view of the dorsal epidermal cells is visible. B: A dorsal view of an *sdn-1(zh20)* animal showing disorganized epidermal cells in the posterior half of the embryo. C: Ventro-lateral view of a wild-type embryo just after ventral enclosure. Asterisks mark the hexagon-shaped lateral seam cells. Note the regular morphology. D: A *sdn-1(zh20)* animal treated with *lin-44 RNAi*. While some lateral seam cells (asterisks) are correctly positioned, others are grossly disorganized (note, some of these seam cell identities were made tentatively, and are based on location). Scale bar = 10 μm.

In wild type animals, AJM-1::GFP can be seen accumulating at cell junctions outlining the epidermal cells, starting around the lima bean stage of embryogenesis, and persisting throughout development (Fig 4). Epidermal cell junctions in wild-type animals are well organized, and only rarely display gaps or misshapen cells. In contrast, we found that both *ptp-3* and *sdn-1* mutants had apparent cell-shape changes consistent with defects in either cell positioning or cell polarity (Fig 4, Table 3).

**Table 3:** AJM-1::GFP analysis by genotype.

		wild-type			*ptp-3*			*sdn-1*		
Class	RNAi	Normal	Defective	% Defective	Normal	Defective	% Defective	Normal	Defective	% Defective
Control	vector	108	0	0%	100	4	4%	13	87	87%
*sdn-1* SynLet	*bli-4*	94	6	6%	12	76	86%	N/D		
*sdn-1* SynLet	*lin-44*	42	81	66%	7	90	93%	N/D		
*sdn-1* SynLet	*oac-9*	22	82	79%	8	93	92%	N/D		
*ptp-3* SynLet	*unc-40*	92	6	6%	4	96	96%	3	99	97%
*ptp-3* SynLet	*vab-1*	35	82	70%	N/D			2	100	98%
*sdn-1 & ptp-3* SynLet	*ncx-4*	94	6	6%	2	98	98%	6	79	93%
Other	*mnp-1*	29	75	72%	0	100	100%	11	91	89%

Interestingly, the presence of disorganized cells as visualized by AJM-1::GFP did not correlate with the embryonic or larval lethality in the various mutant backgrounds assayed. For example, only 4% (4/100) *ptp-3* mutants had obvious defects in the AJM-1::GFP pattern (Table 3). In contrast, 87% (87/100) of *sdn-1* mutants exhibited abnormalities in AJM-1::GFP expression. Despite this, the rate of embryonic lethality in these two backgrounds is similar. Also, when we used RNAi to knock down gene expression in our SynLet screen, we found no correlation between the effect of RNAi on embryonic development and changes in the expression pattern of the epidermal junction marker. Overall this suggests that while these genes may contribute to the positioning of epidermal cells or the organization of epidermal junctions, the proper localization of AJM-1::GFP to these sites is insufficient to explain the lethality observed in the different genetic backgrounds. This is similar to previous reports for cell adhesion molecules in *C*. *elegans*, *e*.*g*. loss of the E-cadherin-like HMR-1, which results in lethality, yet the animals can form and maintain intact epidermal junctions, even when some junctions are failing [67].

### The Wnt ligand LIN-44 functions in gastrulation

One of the strongest SynLet interactions uncovered in our screen was between the Wnt ligand *lin-44* and *sdn-1* (Fig 3, Table 1). Wnts and syndecan have been shown to function together in multiple developmental contexts in both *C*. *elegans* and other systems [43, 46, 67, 68]. The *C*. *elegans* genome encodes five Wnt ligands; *cwn-1*, *cwn-2*, *egl-20*, *lin-44* and *mom-2*. Of these only *mom-2* has been shown to function in embryonic development, as loss of function in *mom-2* results in maternal-effect embryonic lethality [56], although loss of multiple Wnts can result in a more penetrant lethality [69]. In our screen, knockdown of *lin-44* caused a robust increase in the embryonic and larval lethality of *sdn-1* mutants, but not in *ptp-3* or wild-type animals. We did not observe lethality in *sdn-1* animals treated with *egl-20* or *cwn-2* RNAi, while *mom-2* knockdown caused embryonic lethality in all backgrounds tested. *cwn-1* was not present in our RNAi library hence was not assayed.

To better understand the morphogenetic defects behind the *lin-44* and *sdn-1* interaction, we built and analyzed a double LOF line, *lin-44(n1792); sdn-1(zh20)*. *lin-44(n1792)* is predicted to be a complete loss of function mutation at the *lin-44* locus, hence this strain was genetically null for both genes in question. We found that *sdn-1; lin-44* double mutants showed a significant increase in the penetrance of embryonic lethality compared to either single mutant (Table 1).

**Fig 5:**
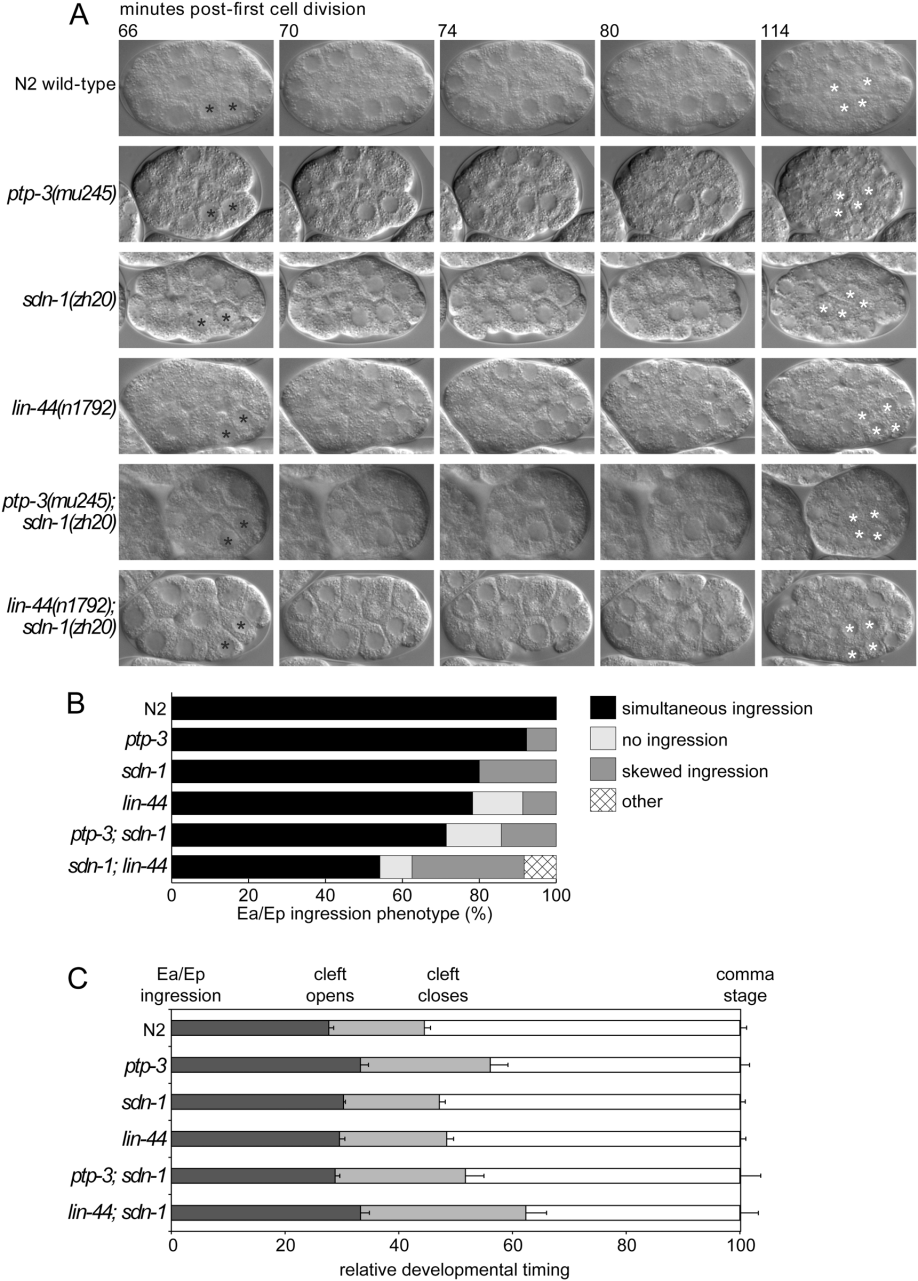
*sdn-1* mutations enhance *lin-44* gastrulation defects. A: In *C*. *elegans*, gastrulation is initiated by the inward migration of the endodermal precursor cells Ea and Ep (black asterisks). In *sdn-1* and *ptp-3* mutant animals, the cells ingress, become completely surrounded by neighboring cells, then divide laterally (white asterisks). Note that Ea and Ep are completely internalized prior to the lateral cell division. In *lin-44* and *sdn-1* mutants, Ea/Ep ingression is often asynchronous. In addition, some *lin-44* embryos show a more severe phenotype, where the Ea/Ep cells completely fail to ingress. The subsequent lateral cell division positions two of the daughter cells onto the surface of the embryo, generating a “Gut on the exterior”, or Gex phenotype [77]. Similar but more penetrant defects are observed in *lin-44; sdn-1* double mutants. B: Summary of Ea and Ep cell ingression behavior by genotype. The *lin-44; sdn-1* double mutants exhibit a higher rate of Ea and Ep ingression defects than either single mutant. C: Relative timing of developmental milestones as a function of genotype. Note the *lin-44; sdn-1* double mutants have a significantly longer period in which the gastrulation cleft is open.

Using time-lapse video microscopy, we found highly penetrant defects in the migration of endodermal precursor cells Ea and Ep, in *lin-44; sdn-1* double mutants at the 24-cell stage of development (Fig 5). In wild type animals, gastrulation begins when Ea and Ep rotate and then ingress from the surface of the embryo to the center [51]. In *lin-44; sdn-1* double mutants, 48% of embryos (11/24) showed defective Ea/Ep ingression, compared to 21% of *lin-44* (5/23) and 20% of *sdn-1* (3/15) single mutant embryos. As a comparison, we also examined *ptp-3; sdn-1* embryos for Ea/Ep ingression failure. 29% (2/7) embryos showed defects in this process. In one embryo, the Ea/Ep cells completely failed to ingress, while in another, Ep ingressed before Ea. These data are not significantly different from *sdn-1* mutants alone, suggesting that *ptp-3* has no obvious role in early gastrulation. These defects in Ea/Ep ingression often resulted in endodermal cells appearing on the embryo’s surface later in development (Gut on the exterior, or Gex phenotype), with catastrophic consequences for subsequent epithelial cell migrations (Fig 5). Further analysis of our time-lapse data revealed that 15% (3/20) of *lin-44* embryos showed defects in neuroblast migration as manifested by an increase in gastrulation cleft duration and failure of epithelial cells to enclose the embryo (Fig 3A and Fig 5). This is likely due to mis-positioned gut cells inhibiting or blocking epithelial cell migrations, or causing defects in overall embryonic organization. While the *lin-44; sdn-1* Ea/Ep ingression phenotypes appear to be additive when compared to each single mutant, the increase in embryonic lethality is clearly synergistic (Table 1). As such, it appears that small defects in Ea/Ep ingression early in development lead to severe consequences in later developmental events. Together these results indicate that both *lin-44* and *sdn-1* contribute to the normal migration of Ea and Ep cells at the onset of gastrulation, and that *ptp-3* has no obvious role in this process. In addition, both *lin-44* and *sdn-1* may also be involved in controlling neuroblast migration and gastrulation cleft closure later in embryogenesis, although in the case of *lin-44*, we cannot rule out that defects at later time points are a consequence of earlier defects in Ea/Ep migration.

In other contexts LIN-44 has been shown to signal through the LIN-17 Frizzled-like receptor. We made double mutants of *lin-17* with *sdn-1*, but found no significant increase in the Emb phenotype compared to *sdn-1* animals alone (Table 1). *lin-17; sdn-1* double mutant adults were strongly Unc, and often died early, around day 2–3 of adulthood, compared to *sdn-1* adults, which live for ~7–10 days (B.D. Ackley unpublished observations). This suggests that LIN-44 affects gastrulation via a different receptor, and does not specifically function via LIN-17.

Finally, we examined whether *ptp-3* loss of function also synergized with *lin-44*. We found that double mutants of *ptp-3(mu245); lin-44(n1792)* exhibited Emb lethality at a rate similar to *ptp-3(mu245)* single mutants (Table 1), although there was a slight increase in larval lethality in the double mutants compared to each single mutant background. This suggests that *lin-44* and *ptp-3* may be functioning in the same genetic pathway during embryonic development. Overall, our results are consistent with *ptp-3* contributing to *lin-44*-dependent embryogenesis, and that at least one of the potential reasons that *ptp-3; sdn-1* double mutants die is because of a disruption in the *lin-44 / ptp-3* signaling pathway.

## Conclusions

Embryonic development requires an orchestrated set of cell migrations and rearrangements, and the proper modulation of cell adhesion is critical to this process. Here we have demonstrated that the type IIa RPTP, *ptp-3* and the syndecan ortholog, *sdn-1*, contribute to parallel genetic pathways during *C*. *elegans* embryogenesis. The dual loss of these molecules results in developmental failure due to defects in two major cellular rearrangements, gastrulation and epiboly. Both *sdn-1* and *ptp-3* have roles at the onset of gastrulation, which is the first major cellular rearrangement seen in *C*. *elegans* development. In addition, both are clearly required for cell migration events later in development at gastrulation cleft closure. Failure to close the gastrulation cleft leads to subsequent defects in hypodermal enclosure and developmental failure via ventral rupture at the onset of embryonic elongation.

At first glance our results seem at odds with data from other organisms, where LAR-RPTPs and syndecans have been found to function as a ligand-receptor pair [33, 34]. In the *Drosophila* neuromuscular junction, cis-interactions on the neural membrane between DLar and syndecan interfere with trans-interactions between neural DLar and muscle-derived glypican. The binding of DLar to syndecan reduces the adhesion at the NMJ and serves to permit expansion of the structure. However, the *C*. *elegans* isoform most similar to DLar, shown to bind syndecan, is PTP-3A, which is genetically and functionally distinct from the isoform we found to cause embryonic defects, PTP-3B. In our assays *ptp-3A* had no effect on embryonic viability, either alone or in combination with *sdn-1*, suggesting that the differences between our observations and others are due to the isoform being analyzed.

The PTP-3B isoform that our data implicate as functioning in parallel to SDN-1 appears to be conserved evolutionarily in vertebrates, but is not obviously found in *Drosophila*. In vertebrates a short isoform of the LAR/PTP-3F receptor has been identified that has the same domain architecture as PTP-3B, being comprised of 5 Fibronectin type III domains in the extracellular portion and two tandem phosphatase domains intracellularly (Uniprot—H0Y4H1). Thus, it is possible that the interaction we have observed between LAR and syndecan is conserved in vertebrates as well.

Our results suggest that in *C*. *elegans* embryonic development, the syndecan and LAR proteins have overlapping roles, and can partially compensate for the absence of each other. Further, we found that animals lacking both *ptp-3* and *sdn-1* exhibited variable points of developmental arrest. This was most apparent in the animals that lacked any maternal *ptp-3* contribution, which arrested embryogenesis during epiboly or at hatching, after completing embryogenesis. The variable arrest points of the *sdn-1; ptp-3* double mutants suggests that there are likely multiple phases of development in which SDN-1 and PTP-3 function in parallel to provide essential functions. However, an alternative hypothesis is that an early defect at the onset of gastrulation can lead to a variable arrest point later in development. Based on our time-lapse analysis, our data favor the former, but it is possible that subtle defects during early development are occurring. To address this point we will need to identify the mechanism(s) that underlie the lethality of the *ptp-3; sdn-1* double mutant animals.

Additional factors likely provide some compensatory function in the absence of both PTP-3 and SDN-1, permitting animals to get through critical developmental periods. For example, some *sdn-1; ptp-3* embryos fail to complete epiboly; if this were the earliest function of PTP-3 and SDN-1, and they could not be compensated for during this process, we would expect 100% of the embryos to arrest at that point in development. However, we find that some animals complete epiboly, but arrest at a later time point. The variable arrest points seen in the double mutants suggest that there are potentially multiple proteins that could partially compensate for the loss of *ptp-3* and/or *sdn-1* during development, including the Eph-ephrin, and slit-robo pathways, which have previously described roles in this process [6, 7, 52].

The SynLet phenotype observed in *ptp-3; sdn-1* double mutants provided us with a powerful platform to identify novel genetic interactions required for embryonic development. We found that we can induce lethality in either the *ptp-3* or *sdn-1* mutant backgrounds by knockdown of orthogonal genes using RNA interference (RNAi). Like most screens using RNAi we observed some variability in the efficacy, compared to known loss-of-function mutations. While this may complicate the interpretation of epistatic relationships, we found RNAi had a robust effect for the purposes of discovery.

Based on the results of our SynLet screen, we propose that there are at least three, and likely more, different cell-adhesion pathways functioning semi-redundantly during *C*. *elegans* development, at least two of which utilize *ptp-3* and *sdn-1*. This is not surprising, as development requires an integrated symphony of cell movements, wherein adhesion must be transiently changed in an orchestrated fashion. However, it is interesting to note that some cell adhesion proteins, *e*.*g*. laminin, perlecan, collagen IV, and integrins are categorically essential to embryonic viability, whereas others, like *ptp-3* or *sdn-1*, have a more flexible requirement. As laminin, perlecan, and collagen IV are all components of the basal lamina, and integrins receptors for some of these proteins, it suggests that the basal lamina is a crucial reference point for cell migrations. Cell adhesion, via proteins like PTP-3B or SDN-1, appears to be a more redundant process, with multiple proteins capable of contributing to this role.

Our SynLet screen has identified multiple genes with previously undiscovered roles in embryonic development. The identification of *mnp-1* lethality being reduced by mutations in *ptp-3* and *sdn-1* lethality suggests a complex interplay between cell adhesion and matrix remodeling proteins during embryogenesis. The Eph receptor *vab-1* has previously been shown to function in parallel with *mnp-1* during *C*. *elegans* embryonic muscle cell migration [66]. *vab-1* also functions in parallel with *ptp-3* to regulate epidermal migration during morphogenesis [5]. The genetic interactions we observe suggest that loss of *ptp-3* was protective to animals where *mnp-1* had been knocked down. Thus, it will be interesting to determine if this reveals a previously unknown role for *ptp-3* in muscle cell migration, or a role for *mnp-1* in epidermal cell migration.

One of the interactions we uncovered using our SynLet RNAi approach was a genetic interaction between *sdn-1* and *lin-44*, one of the five Wnt ligands encoded by the *C*. *elegans* genome [70]. Prior to this, *mom-2* was the only Wnt ligand that had been demonstrated to affect gastrulation. However, it has been shown that other Wnt ligands can influence this process as *cwn-1* and *cwn-2* mutations enhance the *mom-2* lethal phenotype. The fact that the *cwn-1;cwn-2;mom-2* triple mutants exhibit a fully penetrant lethality indicates at least some functional redundancy of Wnt ligands in embryogenesis [69]. *mom-2* contributes both to endodermal specification and gastrulation through partially overlapping functions [56]. Ultimately, MOM-2, functioning through the frizzled-like receptor MOM-5, results in the phosphorylation of myosin light chain to induce constriction of the apical surfaces of the Ea and Ep cells to induce their internalization. Recent work has also uncovered a novel role for SDN-1 in embryogenesis, where it functions in a MOM-2-dependent pathway to control the orientation of the mitotic spindle earlier in embryogenesis (6 to 8 cell stage of development) [71].

Here we find that *lin-44* also affects the internalization of the Ea and Ep cells, albeit to a lesser extent than *mom-2*. The loss of *sdn-1* significantly enhances the penetrance of Ea and Ep ingression defects, and synergistically causes a highly penetrant embryonic lethality. Further, genetic evidence suggests that the LIN-17 frizzled-like receptor does not function in this event, although previous reports have generally found that LIN-44 signals through LIN-17 [72–76].

Previous work suggested that LIN-44 appears to prime cells for other Wnt-signals. For example, in the PLM mechanosensory neurons, LIN-44 activity was required to induce asymmetric localization of LIN-17 which then acted as a receptor for the EGL-20 Wnt ligand [73, 76]. One possibility is that LIN-44 also primes the Ea and Ep cells to respond to the MOM-2 ligand, although this remains to be determined.

The use of parallel genetic backgrounds to identify SynLet interactions allowed us to immediately assign a candidate gene into a genetic pathway, based on the outcome of the screen. For instance, *lin-44* RNAi knockdown strongly enhanced *sdn-1* Lof phenotypes, yet showed no synergistic phenotypes in a *ptp-3* mutant background. This suggests that *ptp-3* somehow functions in the Wnt pathway during *C*. *elegans* embryonic development. The *lin-44; ptp-3* double mutants had an increase in larval lethality over the single mutant backgrounds, it did not result in complete synthetic lethality as nearly 70% of the animals survived to adulthood. This indicates that during larval development, *ptp-3* and *lin-44* may actually function in separate pathways, which contrasts with their linear genetic relationship during embryogenesis. Our future studies will harness *sdn-1* synergistic effects to allow us to further explore the mechanisms by which *lin-44* and *ptp-3* affect gastrulation in *C*. *elegans*.

## Supporting Information

S1 Table Total Results from RNAi screen. S1 Table provides a complete list of the genes targeted by RNAi in our screen, along with the phenotypes observed in the different mutant backgrounds. Because all clones were screened a minimum of two times the phenotypes observed could be from either assay.
(XLSX)
